# Acute megakaryoblastic leukemia arising in a mediastinal mixed germ cell tumor

**DOI:** 10.1002/ccr3.3932

**Published:** 2021-02-18

**Authors:** Alaaeddin Alrohaibani, Philipp W. Raess

**Affiliations:** ^1^ Department of Pathology Oregon Health & Science University Portland OR USA

**Keywords:** acute megakaryocytic leukemia, mixed germ cell tumor

## Abstract

AML, frequently showing megakaryocytic differentiation, is known to arise in MGCT and has a dismal prognosis. Close inspection of MGCT is required to identify concurrent AML.

## Graphical abstract

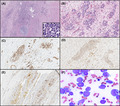

A 30‐year‐old man presented with hemoptysis. A 15 cm anterior mediastinal mass was noted on CT, and a diagnosis of mixed germ cell tumor (MGCT) was made based on core biopsy and elevated serum levels of AFP and β‐hCG. He subsequently received bleomycin, etoposide, and cisplatin at standard dose and schedule. Following the excision of the residual tumor, histologic examination showed a mononuclear proliferation within the MGCT (Figure 1, panel A, 25X). The mononuclear cells infiltrated MGCT tissue and lymphovascular spaces (panel B, 400X). These cells were positive for CD34, CD61, CD117 (panels C‐E, 400X), and dim Factor VIII, and were negative for MPO, CD19, CD3, TdT, and CD68. A diagnosis of acute megakaryoblastic leukemia (AMKL) arising in MGCT was rendered. A bone marrow aspirate and biopsy performed at that time showed morphologically unremarkable trilineage hematopoiesis without flow cytometric or immunohistochemical evidence of involvement by AMKL. Karyotype was normal, and FISH for recurrent genetic abnormalities in AML and targeted high‐throughput sequencing were both unremarkable. The patient was treated with two cycles of a hypomethylating agent. Pretransplant bone marrow biopsy did not show increased blasts but the karyotype demonstrated a near triploid complex abnormal clone. Allogeneic stem cell transplantation was performed and the patient demonstrated engraftment and count recovery at 30 days post‐transplant. However, his platelet counts dropped 3 months after transplant. Imaging demonstrated extensive bilateral supraclavicular, mediastinal and paratracheal adenopathy. A core biopsy of a supraclavicular node and concurrent bone marrow biopsy both showed sheets of megakaryocytic blasts (bone marrow aspirate smear, panel F, 630X) with the same immunophenotype and complex karyotype as seen prior to transplant; prominent RBC and neutrophil emperipolesis was noted in blasts.

**FIGURE 1:**
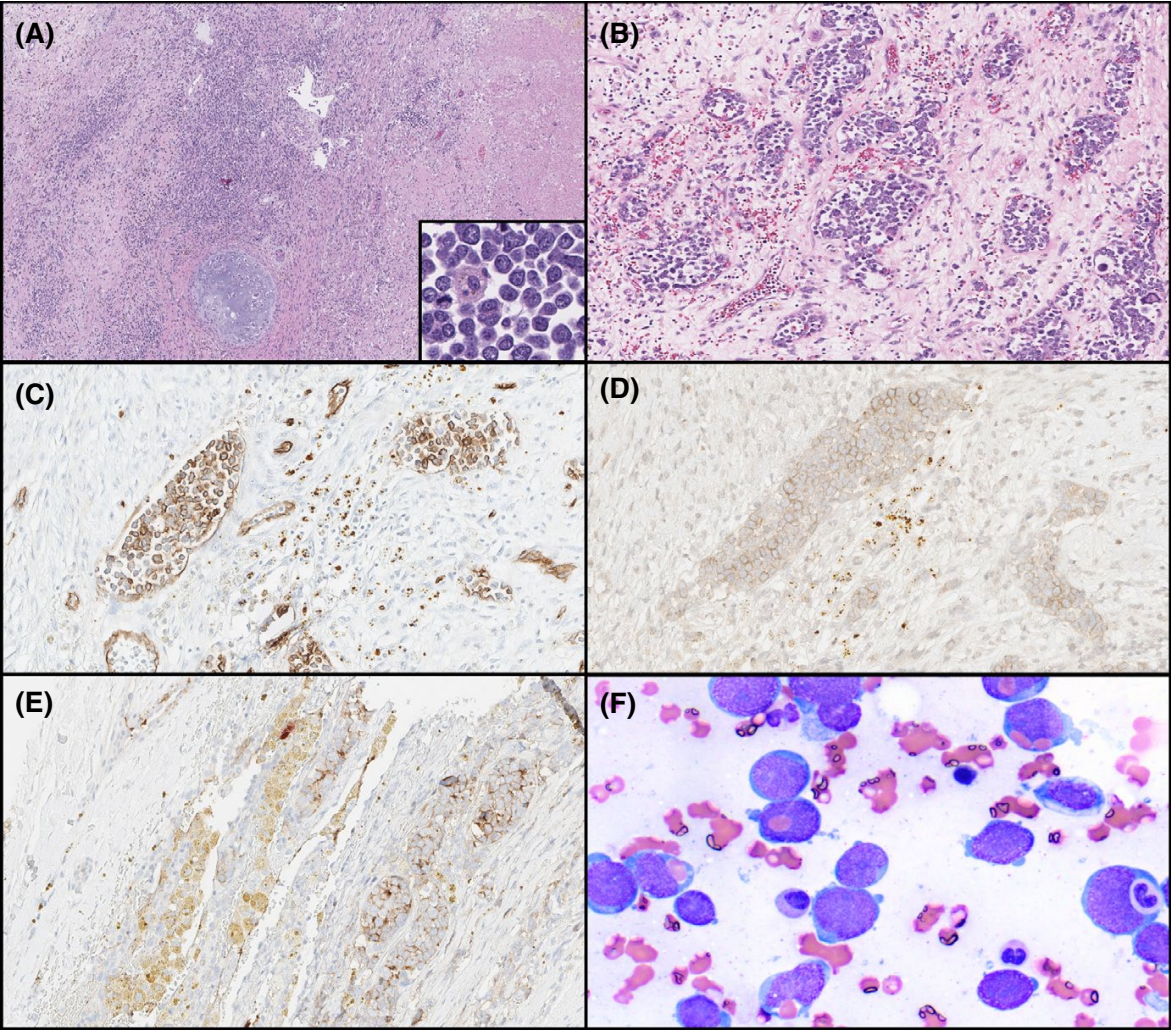
A, H&E photomicrograph showing treated mixed germ cell tumor; inset shows high magnification detail of megakaryocytic blasts. B, blasts are present in lymphovascular spaces. C, CD34 immunohistochemistry highlights blasts. D, CD61 immunohistochemistry highlights blasts and confirms megakaryocytic differentiation. E, CD117 immunohistochemistry highlights blasts. F, Aspirate smear, Wright‐Giemsa stain, highlighting blasts present in bone marrow with emperipolesis of red blood cells and myeloid precursors

## CONFLICT OF INTEREST

The authors state that they have no conflicts of interest.

## AUTHOR CONTRIBUTIONS

AA and PWR wrote the manuscript. AA prepared the figure.

## ETHICAL APPROVAL

IRB approval is deemed not necessary as only one patient is described.

## Data Availability

Data available within the article or its supplementary materials.

